# 3D-Printed Low-Cost Dielectric-Resonator-Based Ultra-Broadband Microwave Absorber Using Carbon-Loaded Acrylonitrile Butadiene Styrene Polymer

**DOI:** 10.3390/ma11071249

**Published:** 2018-07-20

**Authors:** Jian Ren, Jia Yuan Yin

**Affiliations:** 1 Department of Electronic Engineering, City University of Hong Kong, Kowloon, Hong Kong 999077, China; renjianroy@gmail.com; 2 School of Physics and Optoelectronic Engineering, Xidian University, Xi’an 710071, China

**Keywords:** Microwave absorption, dielectric resonator, 3D printing, Acrylonitrile Butadiene Styrene (ABS), ultra-broadband, periodical structure

## Abstract

In this study, an ultra-broadband dielectric-resonator-based absorber for microwave absorption is numerically and experimentally investigated. The designed absorber is made of the carbon-loaded Acrylonitrile Butadiene Styrene (ABS) polymer and fabricated using the 3D printing technology based on fused deposition modeling with a quite low cost. Profiting from the fundamental dielectric resonator (DR) mode, the higher order DR mode and the grating mode of the dielectric resonator, the absorber shows an absorptivity higher than 90% over the whole ultra-broad operating band from 3.9 to 12 GHz. The relative bandwidth can reach over 100% and cover the whole C-band (4–8 GHz) and X-band (8–12 GHz). Utilizing the numerical simulation, we have discussed the working principle of the absorber in detail. What is more, the absorption performance under different incident angles is also simulated, and the results indicate that the absorber exhibits a high absorptivity at a wide angle of incidence. The advantages of low cost, ultra-broad operating band and a wide-angle feature make the absorber promising in the areas of microwave measurement, stealth technology and energy harvesting.

## 1. Introduction

Electromagnetic (EM) absorbers, as the components that allow for EM wave absorption, has aroused significant attention in academia and industry for a long time and is widely used in different areas, such as stealth technology [1,2], EM compatibility and shielding [3], sensors [4,5], energy harvesting [6,7], and passive cooling technologies [8,9,10]. In this context, numerous structures and different materials have been introduced in the design of EM absorbers. The operating frequencies of absorbers have covered the microwave band [11,12,13,14,15], Terahertz band [16,17,18,19,20], and the infrared and visible light band [21]. Different features of EM absorbers, such as their low profile, easy fabrication process and light weight, have been deeply investigated to meet various requirements. Among these features, the low cost and broad operating bandwidth are two pressing issues, as they are highly desired in different applications. In the design of the absorber with a wide operating bandwidth, multilayer structures [1] and taper rod, utilizing high lossy materials [22], are two commonly used methods, which may lead to high cost and bulk volume. In 2008, Landy et al. [23] proposed a new method to obtain ultra-thin microwave absorbers based on metamaterials. The so-called perfect metamaterial absorber (PMA) attracted much attention due to its advantageous low profile and easy fabrication [24,25,26,27]. Due to the resonance property, the operating bandwidth of PMA is usually narrow, which is not enough for certain applications where a wide operating bandwidth is desired. To address this issue, different structures have been introduced to PMA design, such as a tapered helical structure [28], multi-sized tapered waveguide array [29], multiple dielectric layers [30] and a resistance frequency selective surface [31]. Most of these methods utilize a combination of multi-operating bands of PMA. At the same time, it has also been proved that the high absorption can be obtained using materials with high dielectric loss [32,33], indicating that the high-dielectric-loss materials have a large potential to extend the bandwidth of the EM absorbers. Inspired by this fact, materials with different nanoparticles [34] have been investigated to obtain low-cost absorbers with a broad operating bandwidth. In [35], the carbon-coated nickel nanocapsule is proposed for microwave absorption and a maximum reflection loss of 32 dB can be obtained at 13 GHz with 2 mm thickness. ZnO-coated Fe nanocapsules are investigated in [36] for microwave absorption. The reflection loss higher than 20 dB can be obtained in the frequency range of 6.1–15.7 GHz with different thicknesses of the material. In addition, the CaCoTi ferrite composites [37], (Fe, Ni)/C nanocapsules [38], carbon-coated iron nanocapsules [39], Fe_3_O_4_/TiO_2_ Core/Shell Nanotubes [40] and α-Fe_2_O_3_-filled carbon nanorods [41] are also investigated for EM wave absorption at different operating bands. Most recently, composite materials, such as graphene composite [42], α-Fe/Fe_3_C/Woodceramic nanocomposite [43] and Fe_3_O_4_/ZnO core-shell nanocomposite [44], are also studied. Besides, in recent years, the planar metasurface [45], materials with near-zero refractive index [46], emerging graphene [47], spoof surface plasmon polariton [48,49,50] and water [51,52,53,54] are also introduced in the design of a low-cost absorber with a broad operating bandwidth.

At the same time, the emerging additive manufacturing (AM) technology [55,56], often referred to as 3D printing technology, has become a hot area in recent years due to its advantageous low cost, environmental friendliness, and ease of forming. Compared with traditional manufacturing technologies, AM technology can complete irregular profile structures and significantly increase the degrees of freedom for optimal design. Since it was first proposed, different AM technologies have been developed and different kinds of materials have been introduced, such as metal [57] and polymer [58]. The 3D printing technology also has been introduced in the fabrication of different microwave components, such as waveguide [59], lenses [60] and antennas [61]. A 3D printed microwave termination with a tapered shape operating at X-band (8–12 GHz) is proposed in [62]. The 3D-printed absorber based on the filaments with enclosed metal particles is also investigated for microwave absorption at 10 GHz band in [63].

In this paper, a cylindrical-dielectric-resonator-based ultra-broadband absorber for microwave absorption, which is fabricated using 3D printing technology at a low cost, is studied. The carbon-loaded ABS material is introduced in the design of an all-dielectric microwave absorber for the first time. By exciting both the dielectric resonator (DR) mode and the grating mode of the dielectric resonator, near-unity absorption over an ultra-broad bandwidth can be obtained. Benefitting from the frequency-dependent permittivity of the ABS material in the band of interest, the dielectric resonator resonates in its DR mode over broadband at lower frequency with three absorption peaks, corresponding to two fundamental and one higher order magnetic dipole modes. In the higher frequency band, the grating mode of the resonator can be excited, generating one more absorption peak. By optimizing the dimensions of the dielectric resonator, an ultra-broad absorption band can be achieved over the range of 3.9 to 12 GHz with an absorptivity higher than 90%. The operation bandwidth of the designed absorber can reach over 100%. Fabricated by the commercial 3D printer, the prototype of the designed absorber is experimentally investigated, along with full wave simulation. Good agreement between measured and simulated results of the absorber can be observed. Additionally, the angular tolerance of the absorber in transverse electric (TE) and transverse magnetic (TM) mode are both discussed, showing high absorption under wide angles of incidence. All the excellent performance indicate that the designed absorber is promising for different applications, such as radar cross-section reduction, low-cost anechoic chambers, and stealth technology.

The paper is organized as follows: In Section 1, a brief introduction of the work is presented, and the state-of-the-art microwave absorber is given; in Section 2, details of the proposed absorber are presented, including the design diagram of the absorber; in Section 3, the characteristics of the printed material are measured and modeled, followed by numerical simulation of the designed absorber. The working principle of the absorber is discussed in detail in this section. In Section 4, the measurement results are shown and the simulated absorptivity under different angles of incidence is presented. In Section 5, the work is summarized.

## 2. Design of the Dielectric-Resonator-Based Absorber

**Figure 1:**
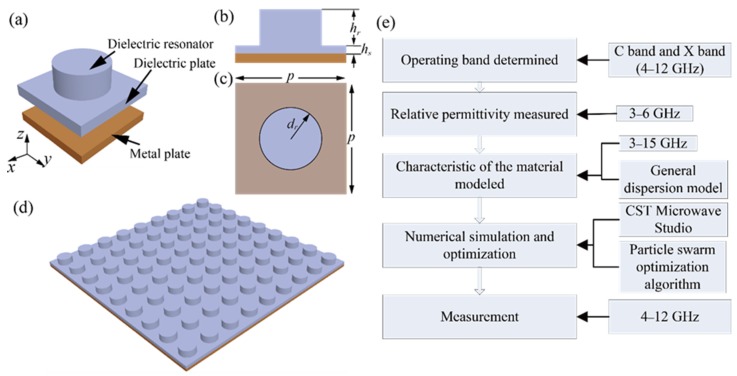
(**a**) Prospective view of the element of the absorber. Each part of the element is ranged layer by layer; (**b**) Side view; and (**c**) top view of the element. The geometrical parameters are as follows: *p* = 23.7 mm, *d_r_* = 7.85 mm, *h_r_* = 6.12 mm, *h_s_* = 3.25 mm; (**d**) Schematic diagram of the whole dielectric-resonator-based ultra-broadband absorber; (**e**) Design diagram and adopted methodology for the design of the absorber.

The perspective view of each single element of the absorber is shown in Figure 1a. The side view and top view of the element are shown in Figure 1b,c, with the dimensions marked. The unit cell is periodically extended in the x and y directions with a lattice period of *p*, forming the whole structure of the absorber, as shown in Figure 1d. Each element consists of two parts, the dielectric resonator and metal backplate. The dielectric resonator is formed with a cylindrical-shaped dielectric body and a dielectric plate, with a thickness of *h_s_*. The cylindrical dielectric body has a radius of *d_r_* and a height of *h_r_*. The commercial 3D printer is used to print the whole structure. The carbon-loaded ABS material, TW-CON175BK, provided by TorwellTechnologies (Shenzhen, China), is used as the dielectric material. The property of the material is measured and modeled, which will be discussed in detail in the next section. The copper plate is used as the metal plate, as shown in Figure 1a. In practice, a copper foil with a thickness of about 0.1 mm is bonded to the dielectric plate’s backside, which will be discussed later. As the metal plate only serves the reflection plate of the absorber, the thickness of the metal plate has little effect on the performance of the absorber if its thickness is much greater than the skin depth of the electromagnetic wave. The skin depth of the copper at 2 GHz is about 0.002 mm, so the copper foil can operate well as the metal plate. The dimensions of the absorber’s element are optimized using the particle swarm optimization algorithm, integrated in Computer Simulation Technology (CST) Microwave Studio to obtain the wide operating band. In the optimization process, the operating bandwidth is set as the optimization goal. In addition, the operating band of the absorber is set to cover the whole C-band (4–8 GHz) and X-band (8–12 GHz), which are widely used in the satellite communication and military applications. Through optimization, good impedance matching can be obtained between the absorber and air, resulting in the incident wave propagating to the absorber and near-unity absorption appearing over a broad bandwidth. The design diagram of the absorber is shown in Figure 1e. The adopted methodology used in each step is also given in this figure for easier understanding.

## 3. Material Characteristic and Numerical Analysis

**Figure 2:**
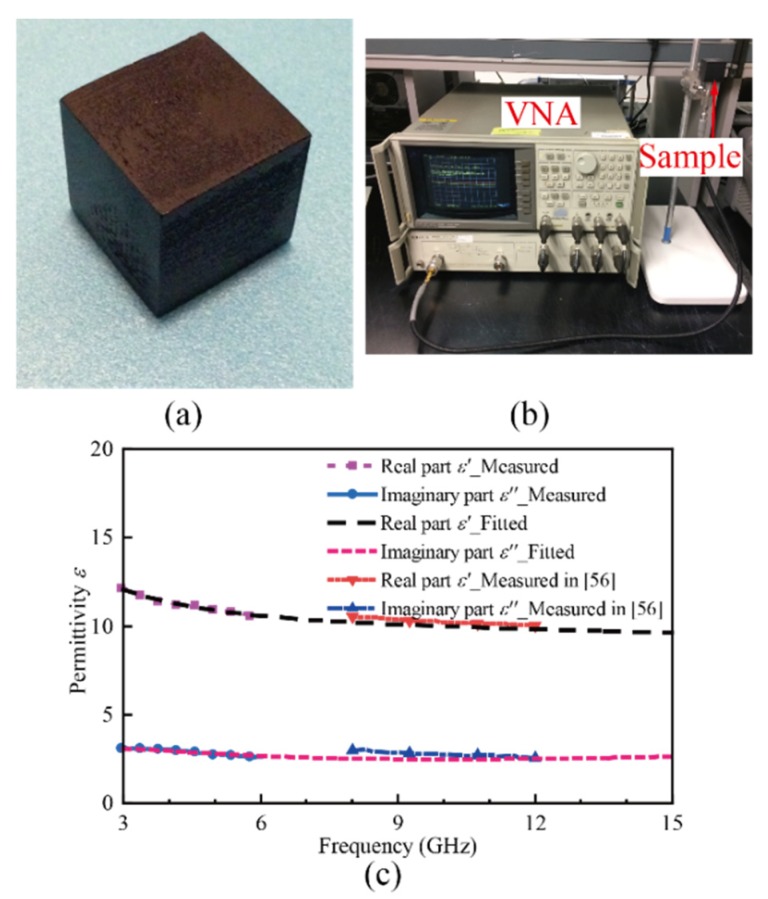
(**a**) Image of the printed cube sample; (**b**) The experimental setup of the permittivity measurement system; (**c**) Measured and fitted data of the permittivity of the material.

To accurately model the designed absorber, the characteristic of the carbon-loaded ABS materials is discussed first. There is previous literature about the carbon-loaded materials for microwave application [64], although the dielectric characteristic of the ABS materials used here is not available. In [62], the same material is used to design a low-cost terminal, and the dielectric constant of the materials at X-band is measured, which is not enough to model the material. To give a comprehensive description of the material, the permittivity measurement system, 85070D Dielectric Probe Kit, provided by Keysight Technologies (California, USA), is used to obtain the data of permittivity. A cube sample with dimensions of a 20 × 20 × 20 mm^3^ is printed using the printer, MakerBot Replicator 2X (Brooklyn, NY, USA), based on Fused Deposition Modeling (FDM) technology. The printer has a nozzle with a diameter of 0.4 mm and the resolution can reach 0.05 mm along the Z axis and 0.1 mm in the XY plane. The printed cube sample and the setup of the permittivity measurement system is shown in Figure 2b. In the measurement system, an open-ended coaxial cable is used as the probe, and the probe is connected to a vector network analyzer (VNA). Before the measurement, a calibration process is carried out to avoid the effect of external noise. The air, a shorted circuit termination and the pure water at room temperature are used as the reference. When the sample is measured, the S-parameter of the probe is measured and then the dielectric permittivity of the material is calculated using measured S-parameter data. The measured real part and the imaginary part of the permittivity are shown in Figure 2c. Limited by the operating frequency of the VNA, we only measured the permittivity from 3 to 6 GHz, and the data corresponding to the rest of the band of interest is predicted using the fitting algorithm, based on general dispersion models integrated into CST Microwave Studio. The predicted data of the permittivity from 3 to 15 GHz are shown in Figure 2c. Additionally, the measured data of the permittivity reported are plotted in the figure. Observing the figure, one can see that there is a good agreement between the data measured in [62] and the predicted data obtained by the fitting algorithm, which can further verify the validity of predicted data. It is also worth noting that, at a low frequency band, i.e., 3–9 GHz, a decreasing trend can apparently be found for the real part of the permittivity. As we all know, for the all-dielectric resonator, the resonating bandwidth suffers narrowband, as the electrical size of the resonator varies with frequency. However, if the permittivity decreases with frequency, this issue may be eliminated, as the electrical length of the structure varies little over a wide bandwidth. This characteristic provides the possibility to design the resonator with a broad operating bandwidth. This will be discussed in detail later. Concerning the data, we also can see that the imaginary part of the permittivity of the materials is maintained at a high level over the whole band, indicating that the dielectric loss of the material is quite high.

After the characteristics of the material are modeled, the numerical analysis, utilizing full wave simulation, is carried out to investigate the working principle of the designed absorber. In the simulation, the element is arranged in the *xoy* plane, and the incident wave is assumed to propagate from the +*z* axis. The unit cell boundary is assigned both in the *x* and *y* axes. For a transmission structure, the absorptivity of the structure can be defined as *A*(*ω*) = 1 − *R*(*ω*) − *T*(*ω*), where *R*(*ω*) and *T*(*ω*) are reflectivity and transmissivity, respectively, which can be calculated using the S-parameters. As the absorber designed here has a metal backplate, the absorption can be calculated with the simplified expression *A*(*ω*) = 1 − *R*(*ω*). Additionally, as the dielectric resonator has a symmetry shape, the absorptivity is independent of the polarization angle of the incident waves. For simplification, only the *x*-polarized incident wave is considered here.

**Figure 3:**
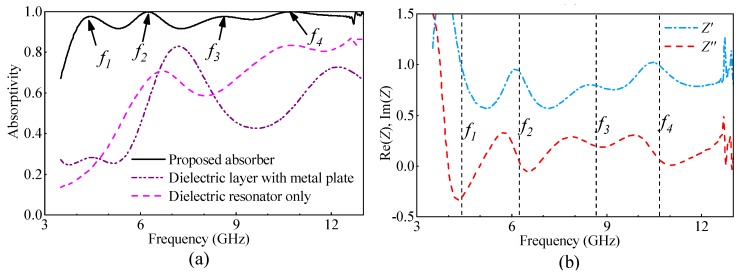
(**a**) Simulated absorption spectra of the designed dielectric-resonator-based absorber. The simulated results for the two comparison cases, namely, the dielectric plate with the metal back and the dielectric resonator without the metal back, are also plotted. The four absorption peaks of the designed absorber are marked as *f*_1_, *f*_2_, *f*_3_, and *f*_4_ in the figure; (**b**) Simulated input impedance of the designed dielectric-resonator-based absorber, with the four absorption peaks marked.

The simulated absorption spectra of the proposed dielectric-resonator-based absorber (black line), under the condition that the incident angle equals to 0°, is shown in Figure 3a. From this figure, one can see that the high absorptivity (higher than 90%) can be obtained from 3.9 to 12 GHz. When the frequency is higher than 12 GHz, apparent ripples can be found in the absorptivity spectra. This is mainly caused by the array diffraction effects of the dielectric resonator, as the lattice constant *p* is comparable to the wavelength. Due to high instability, the absorption higher than 12 GHz is not considered here. In the band from 3.9 to 12 GHz, four peak values can be observed, namely, 4.41, 6.24, 8.61 and 10.76 GHz, which have been marked as *f*_1_ to *f*_4_ in the figure, respectively. The four peak values correspond to four resonating modes. The lower two peak values *f*_1_ and *f*_2_ correspond to the fundamental magnetic dipole mode, or HEM_11*δ*_ mode, of the dielectric resonator, where 0 < *δ* < 1 [65]. The third peak corresponds to the higher order magnetic dipole mode, or HEM_11,1+*δ*_ mode, of the dielectric resonator, while the forth peak corresponds to the grating mode of the dielectric resonator. This will be discussed in detail later. As mentioned above, the dielectric loss of the materials is quite high over the whole band of interest. To give a comprehensive understanding of the merit of the designed absorber, we also simulate two other cases utilizing the same material for comparison. Case I is a dielectric plate with a metal plate backed. In this case, the dielectric plate has a thickness that is the same with the height of the dielectric resonator. Case II consists of the dielectric resonators that are not metal-backed, and the dimensions of the resonator are the same as that of the designed absorber. The simulated results for the two comparison cases are plotted in Figure 3a. From the results, in Case I, the average level of absorptivity is only about 50% over the band of interest, as the permittivity of the dielectric is quite different to that of the air, leading to impedance mismatching at the interface of the dielectric and air. Most of the incident wave is reflected by the dielectric and cannot be absorbed. In addition, two local peak values can be observed at about 7.17 GHz and 12.2 GHz, at which the thickness of the dielectric layer is about 1/2 and 3/4 wavelength, corresponding to the operating frequency. Under these conditions, the dielectric layer can be treated as the impedance matching layer for the incident waves, and good impedance matching can be obtained. However, the bandwidth for the high absorptivity in this case is quite narrow, and the level of absorptivity is low, which is not enough for microwave absorption applications. In Case II, an increasing trend can be observed. Two local peak values can be found in the absorption spectra. These two peak values correspond to the DR mode and grating mode of the dielectric resonator. However, the average level of absorptivity is only as high as 60% and, again, is not enough for microwave absorption applications. To provide further understanding of the designed absorber, the normalized input impedance of the designed absorber is simulated and plotted in Figure 3b. From the figure, we can see that the real part of the input impedance is near-unity, and the imaginary part is near-zero at the four frequencies, *f*_1_ to *f*_4_, indicating that good impedance matching at the interface of the dielectric resonator and air can be obtained at these frequencies, ensuring that the incident wave can propagate into the absorber and then be absorbed entirely as high dielectric loss of the dielectric material.

**Figure 4:**
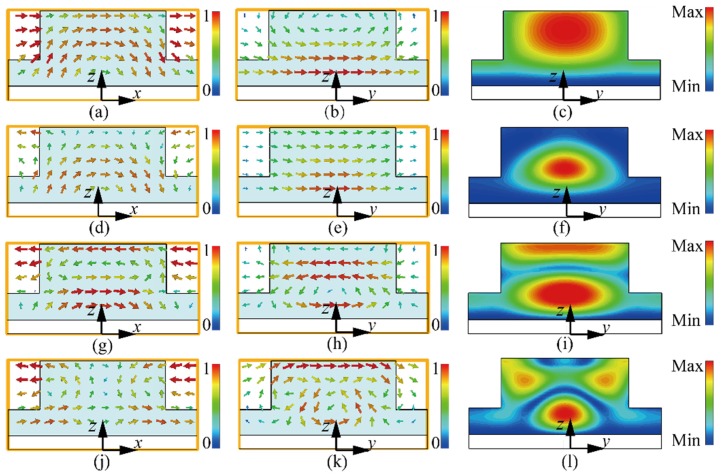
Simulated vector field distribution in the absorber. (**a**,**c**,**e**,**g**) *E*-field in *xoz* plane for *f*_1_, *f*_2_, *f*_3_, and *f*_4_ of 4.41, 6.24, 8.61 and 10.76 GHz, respectively. (**b**,**d**,**f**,**h**) *H*-field in *yoz* plane for *f*_1_, *f*_2_, *f*_3_, and *f*_4_ of 4.41, 6.24, 8.61 and 10.76 GHz, respectively. (**i**,**j**,**k**,**l**) Power loss density of the resonator in *yoz* plane for *f*_1_, *f*_2_, *f*_3_, and *f*_4_ of 4.41, 6.24, 8.61 and 10.76 GHz, respectively.

There are various methods for analyzing the working principle of the absorbers in the literature, such as the effective medium theory [66], transmission line method [67], multi-layer method [68] and scattering theory [69]. These methods are developed for different types of absorbers. The effective medium theory and transmission line method require that the period of the element be much smaller than the wavelength. In the scattering method, the element is treated as the scatterer, and different modes in the element are analyzed. As the period of the element in the absorber proposed here is comparable to the wavelength, the scattering method is used here. The detailed calculation is not the main contribution of this paper, so we only give a qualitative analysis. To deeply investigate the working principle of the designed absorber, the field distribution in the resonator, including the electric field and the magnetic field, at *f*_1_ to *f*_4_ are simulated and plotted in Figure 4. As only the dielectric resonator mode and grating mode are excited, the surface wave [70] is not discussed here. The coordinate system is the same as that shown in Figure 1a. First, the field distribution at frequency *f*_1_ is discussed. In addition, the power loss density in the resonator at different frequencies is also plotted in Figure 4. As shown in Figure 1a,b, a half loop can be observed in the *E*-field, while the *H*-field clearly exhibits a dipole-type-like feature. Considering the resonator is backed with a metal plate, one can include that the fundamental magnetic dipole mode or HEM_11_*_δ_* mode of the dielectric resonator is excited. Observing the field distributions at *f*_2_ shown in Figure 4d,e, we can see that they are quite similar to those at *f*_1_, indicating that the same mode is excited at this frequency. This phenomenon can be explained using the dielectric resonator theory. Giving a dielectric resonator with fixed dimensions, the resonating frequency of the structure can be approximately calculated as *f_r_* = *a*/$\sqrt{\varepsilon_{r}}$, where *a* is the function of dimensions and $\varepsilon_{r}$ is the relative permittivity of the materials used. With reference to the expression, we can see that if the permittivity of the dielectric is constant, the resonator will resonate at a single frequency. However, if the permittivity of the dielectric decreases with the frequency, the structure may resonate over a broad bandwidth, but not at a single frequency. Based on this, it is possible for the magnetic dipole mode to occur at different frequencies, namely *f*_1_ and *f*_2_. As the dielectric resonator mode is excited at these two frequencies, the field is highly confined in the dielectric and absorbed by the material. In addition, observing the power loss density in Figure 4c,f, we also can find that the main loss of the incident power occurs in the cylindrical resonator, which can further prove the excited mode in the dielectric resonator.

Next, the working principle at *f*_3_ is investigated. With reference to Figure 4g–i, a complete and a half loop can be observed in the *E*-field distribution. For the *H*-field distribution, a complete dipole-type distribution can be found at the top part of the resonator, while, at the bottom part of the resonator, a dipole-type distribution with the opposite direction can be observed, indicating that the higher order magnetic dipole mode, or the HEM_11,1*+*_*_δ_* mode, is excited. Additionally, two maximal regions can be found in the figure of power loss density, indicating that the higher order dielectric resonator mode has been excited. Finally, the operating principle at the higher frequency *f*_4_ is discussed. From Figure 4j,k, we can see that the field distribution is quite different from that at *f*_1_ to *f*_3_. In this case, the field is mainly distributed at the gap between the adjacent elements of the absorber. Additionally, the field at the top part of the resonator is strong. This phenomenon indicates that the grating mode of the resonator is excited. By exciting different modes at different frequencies and combining the multi-mode resonances, high absorption over an ultra-broadband can be obtained using the designed absorber.

## 4. Measured Results and Discussion

**Figure 5:**
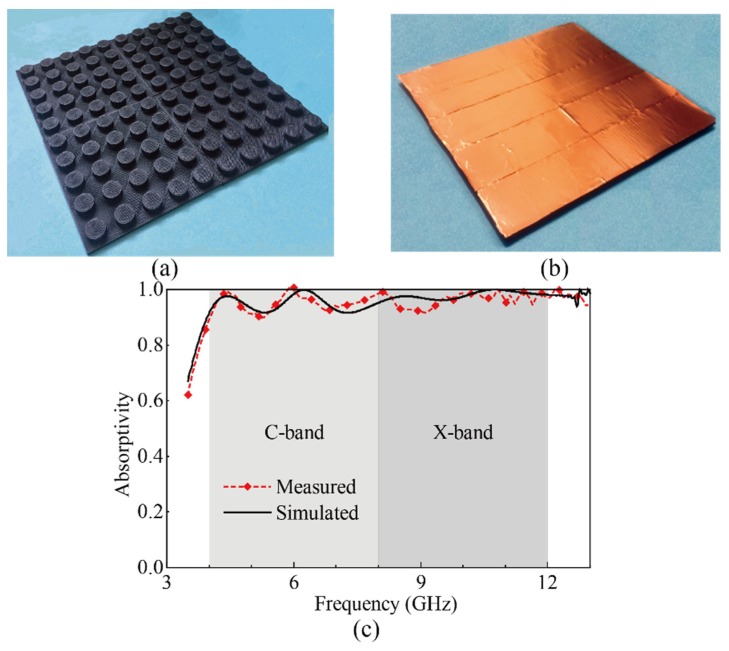
Photo of prototype of the designed absorber. (**a**) Top side; (**b**) Bottom side; (**c**) Simulated and measured absorption spectra of the proposed absorber.

To evaluate the proposed design, the model of the absorber is manufactured and measured. The Prototype of the absorber consists of 10 × 10 elements and the total size of the absorber is 237 × 237 mm^2^. The dielectric resonator is printed using the commercial 3D printer, MakerBot Replicator 2X, based on FDM technology. Limited by the printing size of the 3D printer, the absorber is divided into 2 × 2 substructures, and each substructure consist of 5 × 5 elements. In the printing process, the temperature of the nozzle is set at 230 °C, and the bed temperature is set at 110 °C. The nozzle of the printer has a diameter of 0.4 mm, and the thickness of each layer in the z-axis is set as 0.1 mm, which is the same as that for printing the cube sample. The total time of the printing process is about 24 h. The weight density of the absorber is about 0.55 g/cm^2^, making it suitable for the applications requiring low weight. After the dielectric resonator is printed, a copper foil with a thickness of about 0.1 mm is attached to the back side of the dielectric plate, acting as the metal plate, as shown in Figure 5b. The absorptivity is calculated using the reflection coefficient of the absorber, which is measured using two broadband horn antennas connected to the VNA. To eliminate the effect of the propagation path, the reflection coefficient of a metal plate with the same size as the absorber is also measured and used as a reference. The reflection coefficient of the absorber is normalized to that of the metal plate. The measured results of the absorber are shown in Figure 5c, and the simulated ones are also plotted for comparison. There is a good agreement between the simulation and measurement, and the absorption is higher than 90% in the whole band of interest. There are some variations between simulated and measured results, which may be caused by the imperfections arising from the fabrication process and measurement process. One thing that should be noted is that the incident angle corresponding to Figure 5 is 0°.

**Figure 6:**
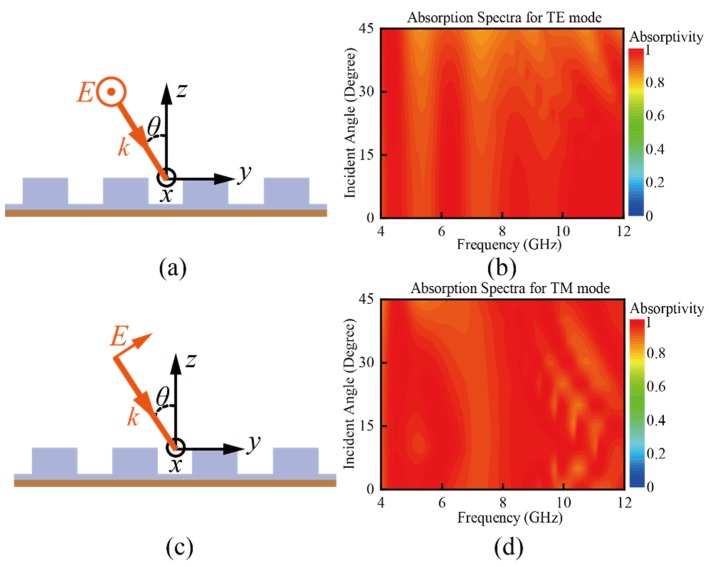
Schematic diagram of oblique-incidence waves for (**a**) the TE mode and (**c**) TM mode. Absorption spectra for oblique-incidence waves with different angles of incidence for (**b**) the TE mode and (**d**) TM mode.

The absorptivities corresponding to the EM waves with different modes and different incident angles are quite crucial for evaluating the performance of the absorber and are also simulated and discussed. The simulated absorptivity of the absorber under different incident angles for transverse electric (TE) mode and transverse magnetic (TM) mode is shown in Figure 6. The incident angles vary from 0° to 45°. From the figure, we can see that in both the TE and TM modes, the absorptivity remains at a high level and the absorption is higher than 90% in most of the band of interest. For the TE mode, when the incident angle increases, the absorption at the lower band decreases, while the absorptivity remains at a high level at the higher frequency band. In the whole band of interest, the absorptivity is higher than 83%. For the TM mode, when the incident angle increases, there is also a decreasing trend that can be found in the absorptivity. However, this trend is inconspicuous compared with that for the TE mode. The absorptivity is higher than 88% in the whole band of interest, indicating that the absorber performs well over a wide range of angles of incidence. The slight difference between the TE mode and TM mode can be explained by the working principle of the absorber, discussed in the last section. For the TE mode, when the incident angle increases, the change of the incident *H*-field is significant, while that of the incident *E*-field is small. As the DR mode excited at the lower frequency band is more sensitive to the changing of the *H*-field, the absorptivity will be much more sensitive to the large incident angle for TE mode, Despite the slight decrease in absorptivity, the absorber exhibits good performance for both the TE mode and TM mode with different incident angles.

## 5. Conclusions

In summary, a microwave absorber with an ultra-broadband operating bandwidth is proposed here. The absorber is made of carbon-loaded ABS polymer material and fabricated by a low-cost 3D printing technology based on FDM. The dielectric characteristic of the printing materials is first measured and modeled in the band of interest. By exciting the fundamental DR mode, higher order DR mode and grating mode of the dielectric resonator, an ultra-broadband operating bandwidth can be obtained. The dimensions of the absorber are optimized using the optimization algorithm and the prototype is fabricated and measured. The simulated and measured results show that, over the whole band from 3.9 to 12 GHz, the absorber exhibits an absorptivity higher than 90%, and the relative bandwidth of the absorber can reach over 100%. The operating band of the absorber can cover the whole C-band and X-band. The working principle of the absorber is discussed in detail with the help of numerical simulation, and the absorptivity under incident angles is also simulated and discussed. As a lightweight microwave absorber with an ultrabroad operation band, the proposed absorber can be used to suppress the radar cross-section of the antenna array, which is useful in military applications [71]. Compared with the metamaterial-based absorber, consisting of the metal wire, the absorber proposed here has a wider operating band and can decrease the complexity of the system. In addition, the absorber can be used in the ultra-wideband (UWB) communications systems to enhance the system’s performance [72,73]. Besides, as the DR mode of the resonator is excited in the absorber, the relative permittivity of the material is significant for the performance of the absorber. As shown above, the relative permittivity of the dielectric used here is about 10. If we choose the lossy material with higher relative permittivity the miniaturization of the absorber can be achieved. In the future, other nanoparticles with extremely high dielectric constants may be considered to enhance the dielectric constant of ABS composite material.
